# The Effect of Low-Fishmeal Feed Including an Olive Leaf Extract (*Olea europaea*) Additive on Growth, Intestinal Health and Disease Resistance in *Litopenaeus vannamei*

**DOI:** 10.3390/ani16152418

**Published:** 2026-08-05

**Authors:** Yundeng Yang, Minling Mao, Huaxing Lin, Beiping Tan, Shuyan Chi, Qihui Yang

**Affiliations:** 1 College of Fisheries, Guangdong Ocean University, Zhanjiang 524088, China; yangyd927@stu.gdou.edu.cn (Y.Y.); 2112201044@stu.gdou.edu.cn (M.M.); 1112201014@stu.gdou.edu.cn (H.L.); bptan@126.com (B.T.); chisy@gdou.edu.cn (S.C.); 2 Guangdong Engineering Technology Research Center of Aquatic Animals Precision Nutrition and High Efficiency Feed, Zhanjiang 524088, China; 3 Guangdong Provincial Key Laboratory of Aquatic Animal Disease Control and Healthy Culture, Zhanjiang 524088, China

**Keywords:** *Litopenaeus vannamei*, plant extracts, growth, intestinal health, disease resistance

## Abstract

This study evaluated the effects of olive leaf extract (OLE) on growth, intestinal health, and resistance against *Vibrio parahaemolyticus* challenge in juvenile *Litopenaeus vannamei*. To a basal diet containing 12% fishmeal, OLE was added at 0%, 0.10%, 0.20%, 0.40%, and 0.80%, forming diets with equal nitrogen and lipid contents. The diets were divided into five groups and named OLE0 (control group), OLE1, OLE2, OLE4, and OLE8. The findings revealed that relative to OLE0, the weight gain rate (WGR) and specific growth rate (SGR) increased in the OLE1 group, whereas the feed conversion ratio (FCR) declined (*p* < 0.05). The crude protein (CP) content for whole shrimp in the OLE8 group was significantly higher (*p* < 0.05), and the crude lipid (CL) content in the OLE1, OLE2 and OLE8 groups significantly decreased compared with OLE0 (*p* < 0.05). In serum indices, the OLE8 group had notably higher total protein (TP) content than the control (*p* < 0.05). Compared with the OLE0 group, high-density lipoprotein cholesterol (HDL-C) levels in the OLE1 and OLE2 groups were increased, whereas low-density lipoprotein cholesterol (LDL-C) levels in the OLE4 and OLE8 groups were significantly decreased compared with the control (*p* < 0.05). Hepatopancreatic malondialdehyde (MDA) in all experimental groups was significantly decreased compared with the control (*p* < 0.05). The superoxide dismutase (SOD) activity in all experimental groups increased (*p* < 0.05) and was the largest in the OLE1 group. The OLE1, OLE2 and OLE4 groups had significantly increased glutathione peroxidase (GPX) activity (*p* < 0.05). Catalase (CAT) activity in the OLE1 group was significantly higher than the control (*p* < 0.05). The *toll*, *lzm*, *imd*, *cru a* and *propo* mRNA expression levels in the hepatopancreas were significantly upregulated in each experimental group (*p* < 0.05). Intestinal microbial analysis indicated that the relative abundance of *Bacteroidetes* was elevated in the OLE4 and OLE8 groups, accompanied by a reduction in *Vibrio* proportion (*p* < 0.05). Post-challenge assays demonstrated that the OLE4 and OLE8 groups achieved remarkably higher cumulative survival rates than OLE0 (*p* < 0.05). In summary, OLE in a low-fishmeal diet can promote growth performance, intestinal health and disease resistance for shrimp. According to the broken-line regression model of WGR against OLE dosage, 0.08% was the optimal dietary OLE inclusion level of the OLE additive in juvenile *L. vannamei*.

## Simple Summary

Fishmeal shortages restrict the sustainable development of shrimp farming, and low-fishmeal feeds often suppress growth and disease resistance of *Litopenaeus vannamei*. Olive leaf extract (OLE) is a natural plant additive rich in polyphenols with antioxidant and immunostimulant activities. In this 8-week feeding trial, five graded OLE supplementation levels (0%, 0.10%, 0.20%, 0.40%, 0.80%) were set in a 12% fishmeal basal diet. Results showed that 0.10% dietary OLE significantly improved growth performance, antioxidant capacity, intestinal digestive enzyme activity and intestinal morphology of shrimp. Higher OLE addition (0.40–0.80%) reshaped intestinal microbiota by reducing pathogenic *Vibrio* abundance and enhanced cumulative survival after *Vibrio parahaemolyticus* infection. Broken-line regression predicted that the optimal OLE supplementation level was 0.08%. This study confirms that appropriate OLE supplementation is an effective strategy to alleviate the adverse impacts of low-fishmeal diets on white shrimp, providing theoretical support for the application of natural plant extracts in shrimp low-fishmeal feed.

## 1. Introduction

*Litopenaeus vannamei*, also known as the white shrimp or White-leg shrimp, is a tropical and subtropical species with wide temperature and salinity tolerances [1,2]. Due to its tender meat and rich nutritional value, it is highly favored by consumers [3,4]. In 2024, China’s *L. vannamei* seawater aquaculture production reached 1.5338 million tons, an increase of 7.27% over 2023, playing an essential supporting role in China’s aquaculture industry [5]. However, with growing scarcity of fishmeal resources, the market price of fishmeal has continued to rise, leading to a significant increase in aquaculture production costs [6]. Against this backdrop, the development and application of reduced-fishmeal aquaculture feed have become a hot topic and a key direction for industry development, posing significant implications for meeting shrimp farming needs. However, reducing fishmeal content in compound feed often leads to adverse effects such as slowed growth rates, decreased survival rates, and weakened disease resistance in farmed shrimp [7]. Accordingly, identifying efficient functional feed additives to mitigate the negative physiological effects of low-fishmeal diets on shrimp has become a core strategy to enhance aquaculture economic returns.

In recent years, many studies have found that Chinese herbal plant additives can both prevent disease and promote growth. Their natural origin, diverse functions, reliable safety, and economic and environmental characteristics make them promising for application in human health [8,9,10]. Furthermore, when used as feed additives, they can promote growth, enhance non-specific immune capacity, and improve antioxidant capacity [11]. Olive leaf extract (OLE) is a natural plant extract derived from olive leaves that serves as a rich source of diverse bioactive compounds [12]. It is rich in olive glycosides, hydroxytyrosol, flavonoids, and chlorogenic acid and possesses multiple functions, including antioxidant, antibacterial, and immune-modulating properties [13]. Currently, existing research has been conducted on the application of OLE in aquaculture animals. In studies on *Cyprinus carpio* and *Oncorhynchus mykiss*, it has been found that OLE can significantly enhance digestive capacity and growth performance [14,15,16]. Additionally, research on Nile tilapia (*Oreochromis niloticus*) and common carp has demonstrated that OLE can remarkably improve antioxidant and immune capacities [15,17]. Among them, there are also relevant studies on shrimp. For example, Gholamhosseini et al. [18] fed 200 mg OLE per kg feed to white spot syndrome virus-infected *L. vannamei*, and the results showed a significant increase in shrimp survival rate. However, the experiment was based on high-fishmeal feed and mainly focused on disease aspects. The effects of OLE on *L. vannamei* under low-fishmeal conditions have not yet been investigated.

Based on the above experiments, further investigations explored the impacts of OLE on *Litopenaeus vannamei* under low-fishmeal diets. Accordingly, this study systematically assessed the comprehensive effects of OLE added to a low-fishmeal diet on non-specific immunity, growth, antioxidant capacity, disease resistance, and intestinal health of *L. vannamei*. This study aims to provide theoretical support for the application of olive leaf extract as a functional additive in low-fishmeal shrimp diets and to promote the transformation of shrimp farming towards a resource-saving, environmentally friendly, and sustainable model.

## 2. Materials and Methods

### 2.1. Experimental Feed Formulation and Feed Preparation

OLE (powder form, 20% oleuropein purity) was purchased from Xi’an Liping Biotechnology Co., Ltd., Shaanxi, China. Details of the feed formulation and nutritional components are shown in Table 1. In the basal diet, OLE was added at 0%, 0.10%, 0.20%, 0.40%, and 0.80%, resulting in five isonitrogenous and isolipids, named OLE0 (control group), OLE1, OLE2, OLE4, and OLE8, respectively.

### 2.2. Experimental Animals and Breeding Management

The trial was authorized by the Animal Ethics and Welfare Committee of Guangdong Ocean University; approval number: GDOU-AEWC-20180063. The trial was conducted in the indoor aquaculture system of the Zhanjiang Marine High-Tech Park of Guangdong Ocean University. Shrimp post-larvae at late post-larval developmental stages were purchased from Zhanjiang Hengxing Zhonglian Aquatic Technology Co., Ltd., and temporarily reared for 30 d, during which time they were fed commercial feed. Feeding was ceased 24 h prior to the formal experiment, when 600 healthy juvenile *L. vannamei* with uniform specifications and an initial body weight of 0.22 ± 0.01 g were randomly selected and stocked into 15 fiberglass tanks with a surface area of 0.3 m^2^ each. This study included 5 treatments, each with 3 replicates, with 40 shrimp per replicate. The entire experimental period lasted 8 weeks, with feeding conducted 4 times daily at 07:00, 12:00, 17:00, and 22:00. The initial feeding rate was set at 8% of the total shrimp weight per tank, and equal feed rations were provided to each tank. The total biomass of shrimp was re-estimated biweekly by random sampling to adjust the daily feeding amount according to residual feed and water quality conditions. Residual feed in each tank was collected 1 h after each feeding, dried to constant weight at 65 °C, and weighed to calculate the actual feed intake per tank. Throughout the trial, the daily feeding amount was adjusted to maintain a small and consistent amount of residual feed across all groups, ensuring that feed availability was not a limiting factor for growth.

### 2.3. Sample Collection

After completing the 8-week farming experiment, *L. vannamei* were subjected to a 24 h starvation treatment, followed by sampling. Each group of tested shrimp was weighed and counted. From each tank, we randomly selected four shrimp and temporarily stored them in a refrigerator. Before sample collection, the shrimp were killed with Tricaine methane sulfonate (MS-222, purity > 95%) [19]. From each tank, we collected blood from the pericardial cavity of four shrimp and temporarily stored the samples at 4 °C overnight (12 h), followed by centrifugation (4 °C, 1191× *g*, 10 min); then, we harvested the supernatant and preserved the samples at −80 °C for later use to determine serum enzyme activity and biochemical indicators. Four shrimp were selected, and the midgut was carefully removed and placed in an explosion-proof tube containing Bohr’s solution for HE (hematoxylin and eosin) staining of the sections. We collected the hepatopancreas and intestines of four shrimp and placed them in explosion-proof tubes, which were quickly placed into liquid nitrogen for cryopreservation prior to further assays. For each replicate, we randomly selected four additional shrimp, removed their hepatopancreas, cut a piece of soybean-sized tissue, placed it in a 1.5 mL centrifuge tube containing RNA-later (Ambion, Thermo Fisher Scientific, Waltham, MA, USA) solution, and incubated it overnight at 4 °C before cryopreservation at −80 °C for relative mRNA expression assessment. Four individuals per replicate were rapidly dissected to collect complete intestines, which were preserved at −80 °C.

### 2.4. Vibrio Parahaemolyticus Challenge Trial

The strains of *Vibrio parahaemolyticus* used in the experiment were obtained from the Key Laboratory of Pathogenic Biology, Fisheries College, Guangdong Ocean University. The *Vibrio parahaemolyticus* strains were cultured in Luria–Bertani liquid medium for 20 h, followed by centrifugation at 7000× *g* for 10 min at 4 °C. We discarded the supernatant and then rinsed the bacterial pellet twice using sterile phosphate-buffered saline (PBS) to eliminate impurities. Afterwards, gradient dilution was performed with PBS to prepare bacterial suspensions at concentrations of 10^9^, 10^8^, 10^7^ and 10^6^ CFU/mL. Prior to the formal challenge experiment, a pre-test was carried out to determine the 4-day median lethal concentration (LD_50_) of Vibrio parahaemolyticus to juvenile *L. vannamei*. Healthy shrimp of uniform size were randomly divided into 5 injection groups and one PBS blank control group; each treatment contained 3 replicates with 10 shrimp per replicate. Each shrimp was injected intraperitoneally with 50 μL of the corresponding bacterial suspension or sterile PBS. Cumulative mortality was recorded every 12 h within 4 days after injection. The LD_50_ value was calculated via the modified Karber method, and the determined 4 d LD_50_ concentration was 1.00 × 10^7^ CFU/mL. During the formal experiment, 50 μL of the bacterial solution at this concentration was precisely injected into the dorsal region of each shrimp’s second to third abdominal somites. After the experiment, the number of surviving shrimps was counted, and the cumulative survival rate (%) was calculated based on this data.

### 2.5. Sample Analysis and Measurement

#### 2.5.1. Feed and Whole Shrimp Proximate Analysis Measurement

Routine proximate composition analyses of feed samples were performed following AOAC official standard methods (AOAC, 1995) [20]. Moisture(MS) of whole shrimp and muscle tissue was determined by vacuum freeze-drying. Feed moisture was measured via the direct drying method at 105 °C. Crude protein (CP) content was quantified using the Kjeldahl procedure. Crude lipid (CL) was extracted by the Soxhlet method using petroleum ether as solvent, and crude ash (CA) was determined after incineration at 550 °C.

#### 2.5.2. Measurement of Serum, Hepatopancreatic, and Intestinal Enzyme Activity

Serum total protein (TP), high-density lipoprotein cholesterol (HDL-C), and low-density lipoprotein cholesterol (LDL-C) levels; acid phosphatase (ACP), alkaline phosphatase (AKP), alanine transaminase (ALT), and aspartate transaminase (AST) activity; maldodicarboxylic acid (MDA) content in the hepatopancreas and the activity of catalase (CAT), superoxide dismutase (SOD), and glutathione peroxidase (GPX); and amylase (AMS), lipase (LPS), and trypsin (TYS) activity in the intestine were assayed via corresponding kits supplied by Nanjing Jiancheng Bio-Research Institute (Nanjing, China), as specified by the kit manuals.

#### 2.5.3. Extraction of RNA from Hepatopancreas Tissue and Real-Time Quantitative PCR Analysis

Initially, RNA was extracted from shrimp hepatopancreatic tissue. Following extraction, 1% agarose gel electrophoresis was used to verify RNA integrity, while ultraviolet–visible (UV-Vis) micro-volume spectrophotometry was applied to test its purity and concentration.

Based on this, RNA was reverse-transcribed into cDNA using the Evo M-MLV RT Mix Kit Ver.2 (EcoRe Bio, Changsha, China). The LightCycler^®^ 480 real-time PCR system was adopted for amplification, and the β-actin gene was chosen as the internal reference gene for relative expression normalization. RT-qPCR for target genes was conducted following the instructions of SYBR Green Premix Pro Taq HS qPCR Kit II (EcoRe Bio, China). Total reaction volume was set to 10 μL, with the following composition: 5 μL 2X SYBR Green Pro Taq HS Premix II mixture (EcoRe Bio, Changsha, China), 1 μL cDNA template, 0.4 μL forward primer, 0.4 μL reverse primer, and 3.2 μL enzyme-free water. Finally, the 2^−ΔΔCt^ algorithm was adopted to compute the relative expressions of target genes [21]. We used specific primer sequences for genes *toll*, *lzm*, *cru a*, *imd*, and *propo* and the reference gene *β*-actin [22] (details of this reference gene are provided in Supplemental Materials Table S1).

#### 2.5.4. Analysis of Intestinal Microbiota 16S rDNA Sequencing

In the present study, intestinal content genomic DNA of *L. vannamei* was harvested; then, the 16S rDNA V3-V4 region was amplified by specific primers. Primers 341F (CCTACGGGNGGCWGCAG) and 806R (GGACTACHVGGGTATCTAAT) were applied in this study. PCR amplicons were purified, quantified, and used for library construction, after which the Illumina PE250 platform was adopted for paired-end sequencing. Bioinformatic analyses were performed as follows: Raw reads were quality-filtered with Trimmomatic v0.33 to discard sequences with an average quality value <20 or ambiguous bases; clean paired reads were merged into tags via FLASH v1.2.11 with a minimum overlapping length of 10 bp. Valid tags were clustered into OTUs at 97% sequence identity using Usearch v10.0, and chimeric sequences were eliminated based on the SILVA v138 reference database. Taxonomic classification of representative OTU sequences was performed against SILVA v138 with a confidence threshold of 0.7 to obtain phylum-to-genus level taxonomic information. To eliminate the interference of uneven sequencing depth across samples, all datasets were normalized to the minimum sample sequencing depth prior to alpha diversity calculation. Indices, including observed OTUs, Ace, Chao1, Shannon and Simpson, were calculated for each normalized sample, and intergroup differences in microbial taxonomic abundance were further analyzed [23].

### 2.6. Formulas and Statistical Analysis

The calculation formula is as follows:

Final body weight (FBW) = *m_t_*;

Weight gain rate (WGR, %) = 100 × (*m_t_* − *m*_0_)/*m*_0_;

Specific growth rate (SGR, %/day) = 100 × (ln *m_t_* − ln *m*_0_)/t;

Feed conversion ratio (FCR) = Dry matter intake (g)/(*m_t_* − *m*_0_);

Survival rate (SR, %) = 100 × Final number/Initial number.

In the formulas, *m*_0_ denotes the initial average body weight (g); *m_t_* refers to the final mean body mass (g); and t stands for the experimental days (d).

One-way ANOVA and Duncan’s multiple range test were conducted via IBM SPSS 22 for intergroup difference analysis. The results are presented as mean ± standard deviation (Mean ± SD). Differences reach statistical significance when *p* < 0.05.

## 3. Results

### 3.1. Effect of OLE on the Growth Performance of Juvenile L. vannamei

**Figure 1:**
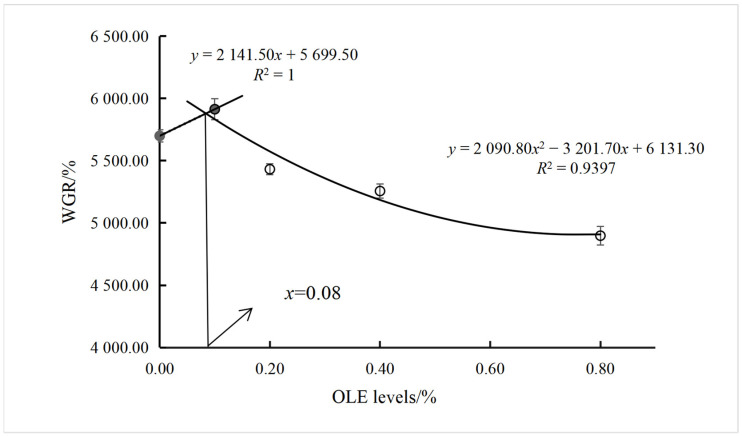
Relationship between OLE levels and WGR in juvenile *L. vannamei* (*n* = 3). The arrow indicates the optimal dietary OLE supplementation level.

OLE had an effect on the growth of juvenile *L. vannamei* (*p* < 0.05, Table 2). FBW, WGR, and SGR reached their maximum values in the OLE1 group and were significantly higher than those in OLE0 (*p* < 0.05). When inclusion levels were ≥0.2%, growth was significantly inhibited relative to the control (*p* < 0.05). FCR was lower in the OLE1 group than in the other groups (*p* < 0.05). No significant effect was observed on SR in each group (*p* > 0.05). Broken-line regression modeling was performed on WGR data from all five experimental groups (OLE0, OLE1, OLE2, OLE4, OLE8). The fitted model estimated that the optimal dietary OLE inclusion level of 0.08% maximized the weight gain rate of juvenile *L*. *vannamei* (Figure 1).

### 3.2. Effects of OLE on the Whole-Body Composition of Juvenile L. vannamei

Relative to the control group, dietary OLE inclusion significantly elevated the CP content in whole shrimp, and the highest value was observed in the OLE8 group (*p* < 0.05, Table 3). CL content in the OLE1, OLE2 and OLE8 groups declined markedly (*p* < 0.05), whereas OLE exerted no remarkable influence on whole-body MS and CA contents (*p* > 0.05).

### 3.3. Effects of OLE on Serum Biochemical Indices in Juvenile L. vannamei

The serum total protein (TP) content in the OLE4 and OLE8 groups was higher than that in OLE0, and the value was highest in OLE8 (*p* < 0.05, Table 4). HDL-C levels were higher in the OLE1 and OLE2 groups compared to OLE0 (*p* < 0.05); LDL-C concentrations declined significantly in the OLE4 and OLE8 groups versus the other groups (*p* < 0.05). The activity of ACP and AKP in the experimental groups increased (*p* < 0.05). OLE significantly reduced AST activity (*p* < 0.05), whereas ALT activity showed no significant variation (*p* > 0.05).

### 3.4. The Effect of OLE on Antioxidant Capacity of the Hepatopancreas in Juvenile L. vannamei

MDA content declined notably in the treated shrimp compared with the control counterpart (*p* < 0.05, Table 5). OLE increased SOD activity in the hepatopancreas (*p* < 0.05), with the highest value observed in the OLE1 group. For GPX activity, no statistical divergence was found between the OLE8 group and the control group (*p* > 0.05); all other experimental groups showed increased GPX activity (*p* < 0.05). The OLE1 group showed significantly increased CAT activity, whereas CAT activity decreased in the OLE2, OLE4 and OLE8 groups (*p* < 0.05).

### 3.5. Effect of OLE on Intestinal Digestive Enzyme Activities in Juvenile L. vannamei

LPS and AMS activities were distinctly elevated in the OLE1 and OLE2 groups versus the control (*p* < 0.05, Table 6), whereas they were significantly reduced in the OLE8 group (*p* < 0.05). OLE at different addition levels significantly increased trypsin activity (*p* < 0.05), but the OLE4 and OLE8 groups presented reduced activities compared with OLE1 and OLE2 (*p* < 0.05).

### 3.6. Effects of OLE on Hepatopancreatic Non-Specific Immune-Related Genes in the Hepatopancreas of Juvenile L. vannamei

**Figure 2:**
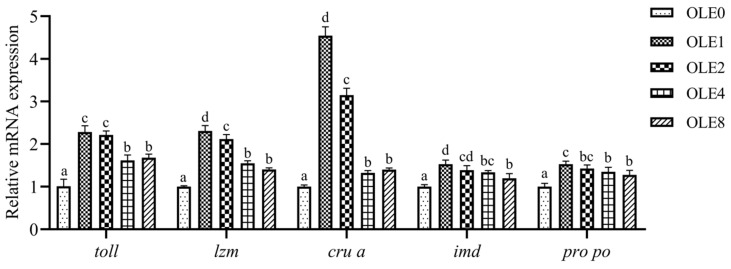
Effects of OLE on hepatopancreatic non-specific immune relative mRNA expression in juvenile *L. vannamei*. There are no significant differences between the values bearing the same superscript letter across groups (*p* > 0.05).

In the hepatopancreas, OLE significantly upregulated the *toll*, *lzm*, *cru a*, *imd*, and *propo* gene transcription levels (*p* < 0.05, Figure 2). All these genes followed a trend of rising first and then dropping (*p* < 0.05).

### 3.7. Effects of OLE on Intestinal Morphology in Juvenile L. vannamei

**Figure 3:**
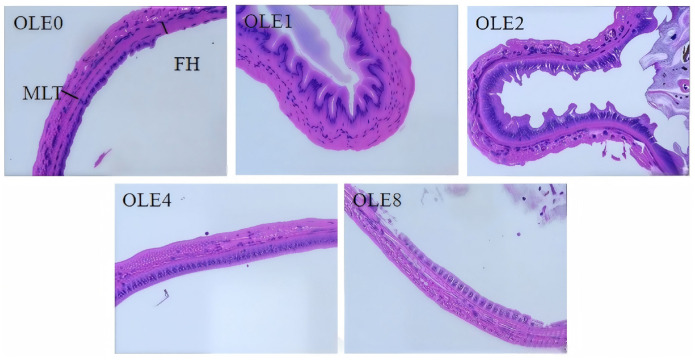
HE sections of the midgut in juvenile *L. vannamei* with different OLE levels.

**Figure 4:**
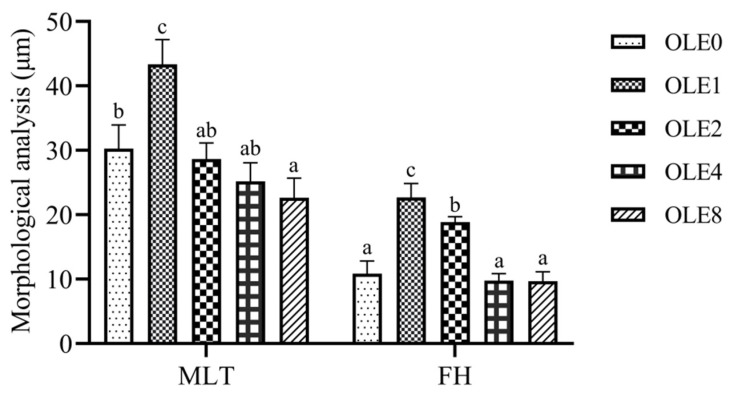
Effects of OLE on the histological analysis of the midgut in juvenile *L. vannamei* (*n* = 3). Abbreviations: MLT, muscle layer thickness; FH, fold height. There are no significant differences between the values bearing the same superscript letter across groups (*p* > 0.05).

The midgut muscular layer thickness markedly increased in the OLE1 group relative to the control (*p* < 0.05, Figure 3 and Figure 4) and then decreased as the OLE concentration increased. No significant difference was observed between the OLE2 and OLE4 groups (*p* > 0.05), whereas the thickness in the OLE8 group was significantly decreased compared with the control group (*p* < 0.05). The OLE1 and OLE2 groups showed significantly increased fold height (*p* < 0.05), while there was no significant difference between the remaining groups (*p* > 0.05).

### 3.8. Effect of OLE on the Intestinal Microbiota in Juvenile L. vannamei

The coverage values of all sample groups exceeded 99.95%, satisfying the requirements for 16S rRNA sequencing; the OLE4 group had a coverage rate of 99.97 ± 0.01% (Table 7). The number of OTUs was significantly higher in the OLE8 group than in the other groups (*p* < 0.05), with no significant differences between the other groups (*p* > 0.05), but the OLE1, OLE2, and OLE4 groups had higher values than the OLE0 group. In terms of α diversity, both the Ace and Chao1 [24,25] indices reached their highest values in the OLE8 group, showing significant differences compared to the other groups (*p* < 0.05). The remaining experimental groups had higher values than the control group, consistent with the trend in OTU numbers. The Shannon index in the OLE1 group was significantly lower than that in the OLE4 and OLE8 groups (*p* < 0.05), but there was no significant difference compared to the control group (*p* > 0.05). The effect of OLE on the Simpson index did not reach statistical significance (*p* > 0.05).

**Figure 5:**
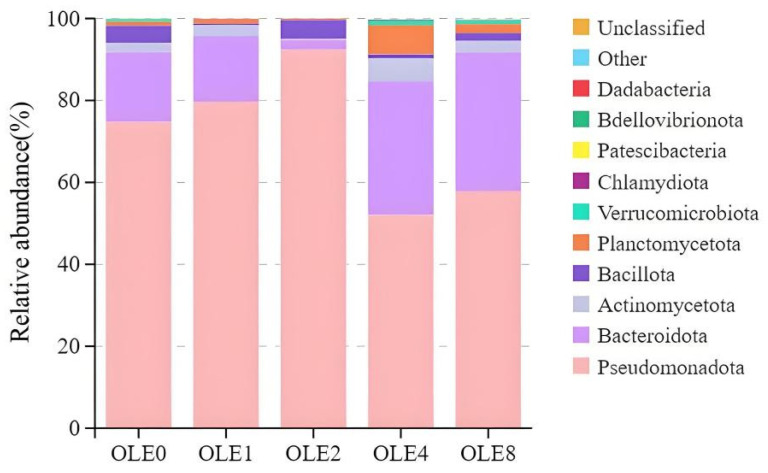
Effects of OLE on the composition of the intestinal microflora in juvenile *L. vannamei* (phylum level, *n* = 3).

From the phylum-level perspective, the dominant bacteria were *Pseudomonadota* and *Bacteroidota*. In contrast, the relative abundance of *Actinomycetota*, *Bacillota*, *Planctomycetota*, *Verrucomicrobiota*, *Chlamydiae*, *Patescibacteria*, *Bdellovibrionota*, *Dadabacteria*, Others, and Unclassified was relatively low. The *Pseudomonadota* phylum abundance in the OLE0, OLE1, and OLE2 groups was higher and lower in the OLE4 and OLE8 groups, whereas the *Bacteroidota* phylum abundance was opposite (Figure 5). Although the values were numerically higher than in the control group, the differences did not reach statistical significance.

**Figure 6:**
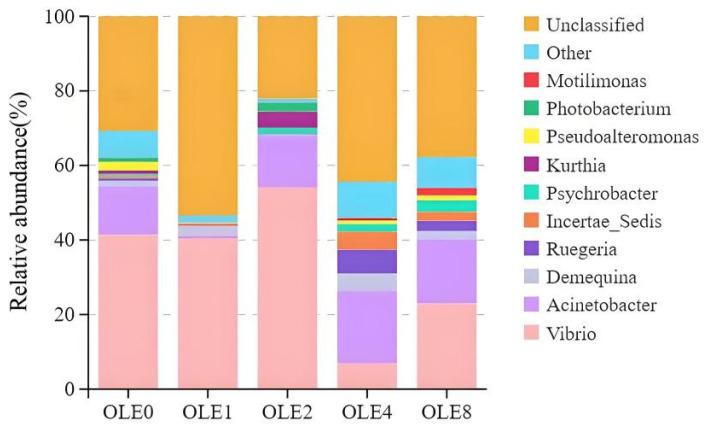
Effects of OLE on the composition of the intestinal microflora in juvenile *L. vannamei* (genus level, *n* = 3).

At the genus level, the dominant bacteria were *Vibrio* and *Acinetobacter*, whereas *Demequina*, *Ruegeria*, *Psychrobacter*, *Kurthia*, and *Pseudomonas* showed relatively low abundance. *Vibrio* abundance in the OLE4 and OLE8 groups was lower than that in the OLE0, OLE1, and OLE2 groups (Figure 6).

### 3.9. Effect of OLE on Disease Resistance in Juvenile L. vannamei

**Figure 7:**
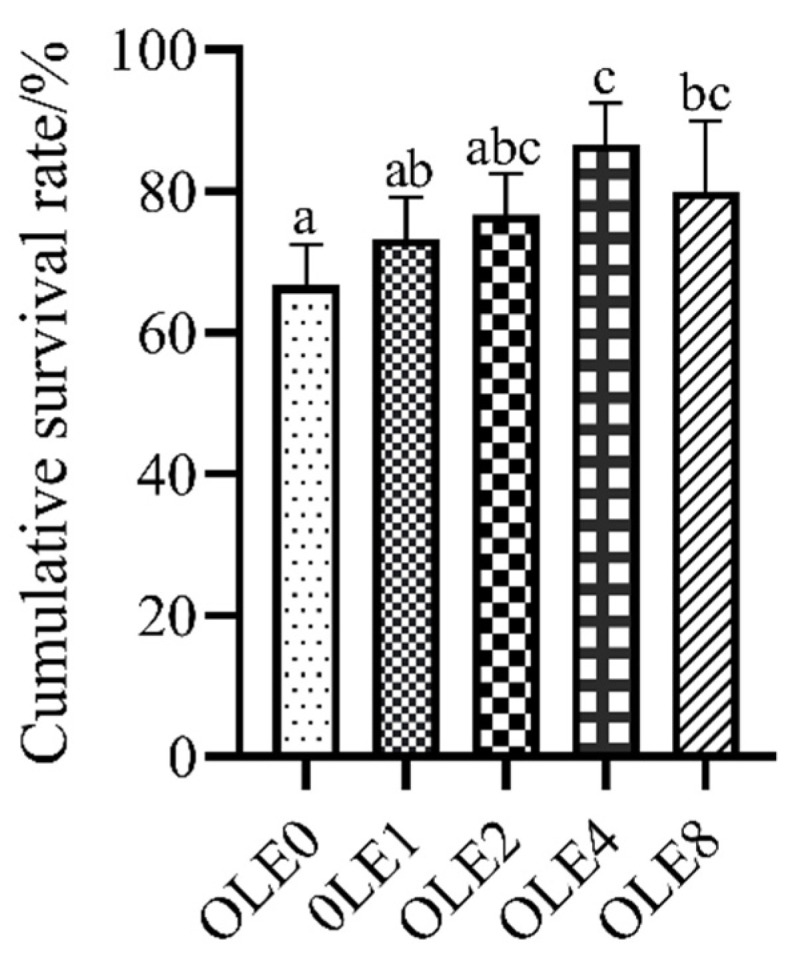
Cumulative survival rate of *L. vannamei* after *Vibrio parahaemolyticus* infection. There are no significant differences between the values bearing the same superscript letter across groups (*p* > 0.05).

Following the *Vibrio parahaemolyticus* challenge trial, the cumulative survival rates of shrimp in the OLE4 and OLE8 groups were significantly higher than those in the OLE0 and OLE1 groups (*p* < 0.05, Figure 7). No significant differences in cumulative survival were detected between the OLE4 group and either the OLE2 or OLE8 group (*p* > 0.05). Similarly, the survival rate of the OLE8 group did not differ significantly from those of the OLE1 and OLE2 groups (*p* > 0.05). No significant difference in post-challenge survival was observed among the OLE0, OLE1 and OLE2 groups (*p* > 0.05).

## 4. Discussion

### 4.1. Effect of OLE on Growth Performance of Juvenile L. vannamei

Currently, numerous studies have demonstrated that OLE has a positive effect on aquatic animals’ growth performance [26]. Hoseinifar et al. [16] reported that adding 0.25% olive pomace cake to rainbow trout feed improved growth performance. Assar et al. [15] also found that the FBW, WGR, and SGR of carp in a 0.10% OLE group were significantly higher than those in other groups, while the FCR was significantly lower than that in other groups. The results of this study are similar to those in previous research; adding 0.10% OLE to the feed significantly improved the FBW, WGR, and SGR of juvenile *L. vannamei*, thereby promoting growth. This result may be closely related to the high content of natural bioactive components such as polyphenols and flavonoids in OLE. Previous studies also suggested that the growth-promoting effect of OLE may be related to phenolic compounds [27], whereas excessive OLE has an inhibitory effect on growth [28]. Xie et al. [29] found that low dietary inclusion of OLE did not adversely affect broiler growth performance, whereas supplementation levels above 0.3% significantly decreased feed intake and inhibited growth. The critical concentration that produced an inhibitory effect was different. The reason may be that the species used in the experiment are different. Besides interspecies discrepancies, inconsistent composition and concentration of bioactive substances in olive leaf extracts from different research also contribute to divergent results [30]. In this experiment, all groups except OLE1 displayed significantly poorer growth performance compared with the control group. Nevertheless, no statistically significant differences in growth indicators were observed among OLE2, OLE4 and OLE8, meaning that growth suppression did not become aggravated linearly with rising OLE inclusion within this dose range. This may be due to the fact that excessive OLE possesses anti-nutritional properties, affecting nutrient absorption in *L. vannamei* [15]. Research has shown that when animals consume excessive amounts of plant extracts, their bodies require additional energy to complete metabolic processes, increasing metabolic burden and ultimately leading to reduced growth performance [31]. Additionally, oleuropein (olive bitter glycoside), the core active component of OLE, has an intense bitter taste. Excessively high dietary OLE levels may impair feed palatability and thereby decrease voluntary feed intake in *L. vannamei*.

In addition, Zemheri-Navruz et al. [27] confirmed that 0.10% OLE can upregulate the expression levels of genes related to growth in carp brain, liver, head kidney, and muscle tissues, thereby improving their growth performance. Previous studies in common carp have shown that OLE polyphenols may promote growth by upregulating growth-related gene expression, modulating antioxidant signaling pathways, and improving nutrient absorption and utilization [15]. Whether the Nrf2/Keap1 pathway is involved in the growth-promoting effect of OLE in *L. vannamei* requires further targeted verification. Therefore, OLE at appropriate concentrations can significantly promote growth performance.

It should be noted that only five discrete OLE inclusion gradients (0%, 0.10%, 0.20%, 0.40%, 0.80%) were established in this trial, and intermediate concentrations such as 0.05% were not included. The 0.08% optimal dosage is a mathematical prediction calculated from the broken-line regression curve rather than an experimentally measured treatment group. Further trials with finer incremental OLE gradients are required to validate this predicted optimal level in the future.

### 4.2. Effect of OLE on the Whole-Body Composition of Juvenile L. vannamei

Research shows that polyphenols and flavonoids contained in OLE have a regulatory effect on the protein and fat content in aquatic animals [18]. Kamboh et al. [32] proposed that flavonoids can enhance the protein synthesis capacity of animal muscles. Olive leaves contain various flavonoid components, such as apigenin, luteolin, rutin, and quercetin [13]. In this experiment, OLE increased the crude protein content in juvenile *L. vannamei*. This is related to the flavonoids contained in OLE.

OLE has a fat accumulation inhibitory effect, and its mechanism of action is relatively complex. Ying Shen et al. [33] found that OLE can effectively alleviate obesity in mice induced by a high-fat diet. The results of this experiment were similar. In the OLE1, OLE2 and OLE8 groups, the crude lipid content of *L. vannamei* was significantly lower than that in the control group. This may be due to the polyphenolic compound oleuropein contained in OLE. Olive glycosides can improve insulin sensitivity, thereby enhancing glucose transport efficiency and preventing fat accumulation caused by hyperglycemia [34]. Although *L. vannamei* cannot produce insulin themselves, insulin signaling pathways and related genes are present in their bodies [35]. Olive glycosides can also alleviate metabolic disorders and reduce weight gain in mice caused by high-fat feed by regulating circulating metabolic factors [36]. From the perspective of molecular gene regulation, oleuropein reduces visceral fat content by upregulating the expression levels of genes mediated by the WNT10B recombinant protein while downregulating the expression of genes related to glycine–methionine peptides [37]. In addition, studies have found that adding oleuropein to a high-fat diet in mice can improve their motor ability and reduce fat cell volume, which may be related to enhanced satiety signals [38]. But these molecular mechanisms have not been validated in shrimp. The consistent lipid-lowering phenotype observed here only reflects a physiological response to OLE supplementation; further transcriptomic analysis is required to uncover the unique lipid metabolism regulatory pathways in juvenile *L. vannamei*. Therefore, OLE may regulate the body composition of juvenile *L. vannamei*, increasing crude protein content and reducing crude fat content. However, further research is needed on the specific mechanisms by which OLE promotes protein synthesis and reduces fat in juvenile *L. vannamei*.

### 4.3. Effect of OLE on Biochemical Indicators in Serum of Juvenile L. vannamei

Protein is the material basis for shrimp growth and maintenance of normal physiological functions, and an increase in total serum protein content reflects an improvement in shrimp health [39]. In this experiment, OLE significantly increased the total protein level in the serum of *L. vannamei*, which was consistent with the body composition results. Previous studies have found that the use of OLE can reduce LDL-C levels and increase HDL-C levels [40]. This experiment also showed that OLE significantly increased HDL-C content while reducing LDL-C content. This improvement in lipid metabolism is closely related to the antioxidant and lipid metabolism-regulating effects of oleuropein and flavonoids in OLE [41]. The increase in HDL-C levels promotes cholesterol reverse transport, reduces abnormal lipid accumulation in the body, and thereby lowers health risks associated with lipid metabolism disorders. Additionally, OLE can optimize lipid metabolism balance by regulating lipid transport enzyme activity, a mechanism similar to its role in reducing the risk of atherosclerosis [42]. However, the specific regulatory pathway of OLE in shrimp lipoprotein metabolism remains to be elucidated, given the substantial differences in lipid transport systems between crustaceans and mammals.

ACP and AKP are key enzymes in the non-specific immune system, and their enhanced activity indicates improved immune function in shrimp [43]. OLE significantly enhances the immune defense capacity of *L. vannamei* by regulating the activity of these enzymes. In this experiment, OLE reduced AST activity. AST is a commonly used indicator for hepatopancreas cell damage, and a reduction in its activity usually means that the degree of liver cell damage has been reduced [44]. Therefore, OLE has a significant improving effect on serum biochemical indicators in juvenile *L. vannamei* through its active ingredients, which can enhance immune function, optimize lipid metabolism, protect hepatopancreas function, and thereby promote shrimp health.

### 4.4. Effect of OLE on the Antioxidant Capacity of the Hepatopancreas in Juvenile L. vannamei

The hepatopancreas is a key metabolic and detoxification organ in crustaceans, and its antioxidant capacity directly reflects the regulatory effect of exogenous substances on the redox balance of an organism [45]. Assar et al. [15] demonstrated that inclusion of 0.10% OLE enhanced carp’s antioxidant function by increasing SOD and GPX activity and reducing MDA content. In laying hens, OLE has also been observed to reduce MDA content [46]. The decrease in MDA levels in this experiment may be attributed to the direct scavenging of free radicals by polyphenolic compounds in OLE [47]. Polyphenolic compounds exert their antioxidant effects by providing hydrogen atoms or transferring electrons to convert free radicals into stable substances [48].

SOD and GPX activities in a living organism’s liver are related to their antioxidant capacity. In *Oncorhynchus mykiss*, Hoseinifar et al. [16] demonstrated that a group including 0.25% olive pomace cake exhibited the highest liver SOD and GPX activities, while the control group exhibited the lowest. In this research, the SOD activity in the OLE1 group reached its maximum value, which may indicate that the activation effect of OLE on SOD is most significant at this addition level. The promoting effect of appropriate OLE supplementation on SOD activity in the hepatopancreas may be attributed to the phenolic compounds in OLE, which scavenge excess reactive oxygen species and alleviate oxidative burden, thereby reducing the consumption of endogenous SOD. Alternatively, bioactive components in OLE can regulate key antioxidant transcriptional pathways to upregulate the expression of SOD-encoding genes, jointly enhancing the antioxidant capacity of the hepatopancreas, suggesting the existence of an optimal dose range that can maximize its antioxidant effects. Notably, although the Nrf2/Keap1 axis is recognized as a core antioxidant regulatory pathway in vertebrates [49], the antioxidant transcriptional network of crustaceans has undergone substantial evolutionary divergence. The enhanced antioxidant phenotype observed in this study may result from either direct free radical scavenging by OLE polyphenols or transcriptional regulation of antioxidant enzymes; however, whether the Nrf2/Keap1 pathway is involved in this process in shrimp remains unconfirmed and requires targeted molecular verification. The GPX activity enhancement by OLE further confirmed its positive effect on enhancing hepatopancreas antioxidant capacity in *L. vannamei*, whereas the OLE8 group did not show significant differences, which may be related to the regulatory effect of OLE on GPX at this concentration or interference by other factors. Furthermore, there are differences in the regulatory effect of OLE on CAT at different concentrations, and it may even have an inhibitory effect. In the OLE1 group, CAT activity was significantly increased, whereas in the OLE2, OLE4, and OLE8 groups, CAT activity was significantly decreased. The increase in CAT activity in the OLE1 group may be related to its synergistic action with SOD and GPX, which enhances antioxidant defense capabilities. Pan Yao et al. [50] pointed out that although polyphenolic compounds possess antioxidant properties, at high concentrations, they compete with other antioxidant components for reaction sites, thereby weakening overall antioxidant efficacy. Therefore, in practical application, the dosage and complexity of OLE’s action mechanism need to be considered. Future research can further explore the effects of OLE on other antioxidant-related indicators and its mechanism of action under different physiological and environmental conditions, providing more solid data for the cultivation and health protection of *L. vannamei*.

### 4.5. Effect of OLE on the Expression of Non-Specific Immune Genes in the Hepatopancreas of Juvenile L. vannamei

Previous studies have shown that plant extracts can regulate the expression of immune-related genes in aquatic animals [51]. In this research, OLE exhibited significant regulatory effects on *toll*, *cru a*, *imd*, *propo*, and *lzm* mRNA expression, with peak expression levels observed in the OLE1 and OLE2 groups. This may be due to the fact that OLE is rich in active ingredients such as polyphenols, which activate innate immune signaling pathways and enhance immune effector molecule expression, thereby influencing the mRNA expression of immune-related genes in the body [52]. Toll and IMD cascades are conserved core modules of innate immunity across metazoans [53]. In vertebrate models, polyphenols such as oleuropein have been found to activate innate immunity via pattern recognition receptors [54]. In the present study, the upregulation of *toll* and *imd* transcription may indicate a similar immunomodulatory pattern, but the specific activation mechanism of OLE on the shrimp Toll/IMD pathway remains to be confirmed at the protein and cellular levels. Whereas the enhanced expression of *propo* and *lzm*, as precursors and key factors of immune effector molecules [55], further confirms the comprehensive activation of the immune defense system by OLE. In this experiment, the *cru a* gene was significantly upregulated. It can play a key role in pathogen recognition through its encoded chitin-binding protein [56]. This is consistent with the findings that the *cru a* gene is significantly upregulated after *Palaemon carinicauda* infection with *V. parahaemolyticus*, indicating that it plays an important role in the innate immune response [57].

Research has found that the combined use of OLE and florfenicol can reduce the gene expression levels of inflammatory cytokines TNF-α and IL-1β in rainbow trout and upregulate the expression levels of antimicrobial peptide genes [58]. However, in this research, when the addition amount exceeded 0.20%, the expression levels of each gene showed a downward trend relative to OLE1 and OLE2. This non-linear dose effect may suggest the existence of a complex regulatory balance mechanism in juvenile *L. vannamei*. In addition, certain components in plant extracts may produce antagonistic effects when concentrations exceed critical levels [50]. Although OLE exhibits significant immune-enhancing effects, excessive inclusion may have the opposite effect. Future studies should combine proteomics and metabolomics approaches to thoroughly analyze the molecular networks involved in the formation of dose–response curves while also exploring the synergistic effects of OLE with other immune enhancers, thereby providing a theoretical basis for the development of precision immune modulators.

### 4.6. Effects of OLE on Intestinal Enzyme Activity and Morphology in Juvenile L. vannamei

Studies have shown that appropriate feed additives can improve the digestive and absorption functions of aquatic animals by affecting digestive enzyme activity and intestinal morphology and structure [59]. Zemheri-Navruz et al. [27] showed that adding 0.10% and 0.25% OLE to feed can significantly enhance carp amylase, protease, and lipase activities. In studies on *Monopterus albus*, grape seed and artemisia extracts can enhance digestive enzyme activity, but excessive addition may inhibit enzyme activity, primarily due to the high polyphenol content in the extracts [60]. OLE itself also contains various phenolic compounds. In this experiment, the addition of OLE had a first increasing, then decreasing effect on the intestinal lipase and amylase activities in juvenile *L. vannamei*, which may be due to its regulation of digestive enzyme secretion through specific mechanisms. Vyas et al. [61] believe that when the amount of plant active ingredients is excessive, they bind to the active sites of digestive enzymes, thereby interfering with their normal function. This inhibitory effect is mainly achieved through hydrophobic interactions and hydrogen bonds. Previous studies have found that adding 0.1% OLE to feed significantly increased trypsin activity in Cyprinus carpio. The results of this experiment are consistent with theirs [27]. The reason for this may be that OLE regulates the intestinal microenvironment and promotes its activity.

OLE possesses significant antioxidant capacity, alleviating oxidative stress on intestinal mucosa, protecting epithelial cells from free radical damage, and maintaining intestinal structural integrity [15]. Research has shown that 10% olive pomace can increase the height, surface area, and ratio of duodenal villi in broiler chicken, thereby optimizing intestinal morphological characteristics and enhancing digestive and absorptive capacity [62]. Another study also found that adding 0.15% olive pomace extract to broiler chicken diets not only did not affect growth performance but also reduced duodenal crypt depth [63]. In this experiment, the addition of 0.10% OLE significantly increased *L. vannamei*’s midgut muscle layer thickness and fold height. Nevertheless, the histological structure and cell composition of the shrimp midgut are fundamentally different from the avian small intestine. The morphological improvement observed in the two taxa is a parallel phenotypic response, but the specific cellular and molecular mechanisms driving this change are likely distinct between crustaceans and birds.

One of the important causes of damage to the intestinal mucosal barrier function is oxidative stress [64]. However, when the addition level equaled or exceeded 0.20%, this study observed a significant reduction in midgut muscle layer thickness and fold height in shrimp, with the muscle layer thickness in the OLE8 group significantly lower than that in the control group. The reason may be that phenolic substances at high concentrations act as reducing agents in the Fenton reaction to generate ROS, triggering oxidative stress. At this point, the role of phenolic compounds shifts from antioxidant to prooxidant, leading to abnormalities in the structure and function of the intestinal mucosa, manifested as muscle layer thinning and fold atrophy [65]. Therefore, the appropriate amount of OLE can be added to improve the digestive enzyme activity (especially trypsin) in juvenile *L. vannamei* and improve the midgut’s morphological structure (increase muscle thickness and fold height), thereby improving their digestive and absorption capacity.

### 4.7. Effects of OLE on the Intestinal Microbiota in Juvenile L. vannamei

Feed components have a significant impact on aquatic animals’ intestinal flora structure and function and affect their survival and growth [66]. The core microbial communities in the intestines of *L. vannamei* are Proteobacteria, Bacteroidetes, Actinobacteria, and Firmicutes [67]. This study obtained similar results, with *Pseudomonadota* (the new name for the Proteobacteria after taxonomic revision), Bacteroidetes, Actinobacteria, and Firmicutes dominating. An increase in *Pseudomonadota* abundance is associated with an increased risk of disease [68]. In this experiment, a significant decrease in the *Pseudomonadota* phylum relative abundance and a significant increase in the Bacteroidetes phylum were observed in the OLE4 and OLE8 groups, indicating that higher dietary OLE levels improve the disease resistance of *Litopenaeus vannamei*; this beneficial effect is likely achieved via reshaping the intestinal microbiota. Bacteroidetes can inhibit pathogenic bacteria colonization by secreting antimicrobial substances, competing for nutrients, and occupying ecological niches in the intestine, thereby maintaining the balance and microbial community health [69].

*Vibrio* is a common dominant bacterial genus in marine environments and one of the main pathogens of marine aquaculture animals [70]. This study showed that increasing the level of OLE added significantly reduced the relative abundance of *Vibrio* species and effectively inhibited pathogenic *Vibrio* proliferation. However, in this experiment, high levels of added OLE inhibited the growth of *L. vannamei*. Some studies suggested that intestinal flora health status may not be directly reflected in host growth performance. Although diversity and intestinal flora stability are closely related to host health, the relationship between the two is influenced by many factors. For example, certain changes in the gut microbiota may enhance the host’s immune function or disease resistance, but these changes do not necessarily directly improve growth [71]. The relationship between the two needs to be further explored in combination with the OLE additive dosage and host physiological characteristics.

### 4.8. Effect of OLE on Disease Resistance in Juvenile L. vannamei

Research on the disease resistance properties of OLE has been conducted in the aquatic animal field, and it plays an important role in the disease resistance field [72]. Some studies have pointed out that adding 0.10% OLE can enhance carp resistance to *Edwardsiella* and improve survival rates [27]. Assar et al. [15] revealed that OLE enhanced disease resistance in Aeromonas hydrophila-infected common carp, which was associated with modulation of the Nrf2/Keap1 antioxidant signaling pathway. In this study, high-dose OLE supplementation also improved the cumulative survival rate of *L. vannamei* after *V. parahaemolyticus* challenge, showing a consistent disease-resistant phenotypic effect. However, the crosstalk between antioxidant signaling and immune defense in crustaceans is not identical to that in teleost fish. The improved survival observed here cannot be directly attributed to the same Nrf2-mediated regulatory mechanism reported in carp. The specific immune-enhancing pathway of OLE in shrimp requires further exploration. Shiry et al. [58] discovered that an OLE and florfenicol combination can enhance the protease and LZM activities in rainbow trout infected with *Streptococcus iniae*. Gholamhosseini et al. [18] studied *L. vannamei* infected with white spot syndrome virus and found that feeding them 0.02% OLE for 7 d significantly improved their survival rate, indicating that OLE helps prevent and control white spot syndrome in shrimp. Additionally, Baba et al. [73] found that adding 0.10% OLE to feed could reduce mortality in *O. mykiss* infected with *Yersinia ruckeri*. The results of the *V. parahaemolyticus* challenge test showed that the shrimp in the OLE4 and OLE8 groups had significantly improved cumulative survival rates. This result was consistent with the decrease in the relative *Vibrio* abundance at the genus level in the intestinal microbiota of these two groups. This may be due to the antimicrobial effect of oleuropein, which has antibacterial properties in OLE, by interfering with the bacterial growth cycle, especially delaying or inhibiting their logarithmic growth phase [74]. OLE may exhibit disease resistance potential through a multi-target mechanism (antioxidant, non-specific immunity enhancement, antibacterial and antiviral, etc.) and can effectively promote the healthy growth of *L. vannamei*. Notably, relatively high inter-replicate variation in survival data limited the statistical separation of the OLE2, OLE4 and OLE8 groups. Further trials with more biological replicates and finer OLE dosage gradients will be conducted to more accurately determine the optimal OLE inclusion level for enhancing resistance against Vibrio parahaemolyticus infection.

## 5. Conclusions

An appropriate amount of OLE in low-fishmeal feed can promote the growth and antioxidant capacity of *L. vannamei*, improve non-specific immunity and intestinal digestive enzyme activity, improve intestinal health and enhance survival against *Vibrio parahaemolyticus* challenge. Based on the broken-line model of WGR corresponding to OLE levels, the optimum addition level of dietary OLE was 0.08% in juvenile *L. vannamei*.

## Figures and Tables

**Table 1:** Composition and nutrient levels in basal diets (% dry matter).

Ingredients	Groups				
	OLE0	OLE1	OLE2	OLE4	OLE8
Soybean meal	21.00	21.00	21.00	21.00	21.00
Brown fishmeal	12.00	12.00	12.00	12.00	12.00
Corn gluten meal	8.00	8.00	8.00	8.00	8.00
Peanut meal	9.00	9.00	9.00	9.00	9.00
Shrimp shell powder	6.00	6.00	6.00	6.00	6.00
Beer yeast	6.00	6.00	6.00	6.00	6.00
Wheat flour	24.00	24.00	24.00	24.00	24.00
Phospholipids	1.30	1.30	1.30	1.30	1.30
Fish oil	1.50	1.50	1.50	1.50	1.50
Soybean oil	1.50	1.50	1.50	1.50	1.50
Ca(H_2_PO_4_)_2_	1.50	1.50	1.50	1.50	1.50
Choline chloride	0.50	0.50	0.50	0.50	0.50
Vitamin C	0.05	0.05	0.05	0.05	0.05
Vitamin and mineral premix ^a^	1.00	1.00	1.00	1.00	1.00
*L*-lysine	2.29	2.29	2.29	2.29	2.29
*DL*-methionine	0.92	0.92	0.92	0.92	0.92
OLE	0.00	0.10	0.20	0.40	0.80
Microcrystalline cellulose	6.12	6.02	5.92	5.72	5.32
Total	100.00	100.00	100.00	100.00	100.00
Nutrient level ^b^					
Crude protein	38.39	38.49	38.52	38.38	38.43
Crude lipid	6.66	6.72	6.60	6.64	6.79
Crude ash	9.60	9.45	9.57	9.52	9.56
Moisture	9.98	9.32	9.15	10.33	10.30

^a^ Vitamin and mineral premix (per kg): Retinyl acetate 450,000 IU, vitamin D_3_ 100,000 IU, *DL*-α tocopherol acetate 5.00 g, pyridoxine hydrochloride 0.60 g, Menadione 0.50 g, Thiamine Nitrate 0.50 g, Riboflavin 0.70 g, Nicotinamide 3.50 g, Cyanocobalamin 0.002 g, *D*-calcium pantothenate 2.00 g, folic acid 0.15 g, *D*-biotin 0.006 g, *L*-ascorbate-2-phosphate 10.00 g, Inositol 8.00 g, Mg 20.00 g, Zn 7.50 g, Mn 2.00 g, Fe 2.00 g, Cu 1.50 g, Co 0.08 g, I 0.10 g, and Se 0.01 g. ^b^ Measured values.

**Table 2:** Effects of OLE on growth performance of juvenile *L. vannamei*.

Items	Groups				
	OLE0	OLE1	OLE2	OLE4	OLE8
FBW/g	12.76 ± 0.11 ^d^	13.23 ± 0.18 ^e^	12.17 ± 0.10 ^c^	11.78 ± 0.12 ^b^	11.00 ± 0.17 ^a^
WGR/%	5699.49 ± 48.13 ^d^	5913.64 ± 82.95 ^e^	5431.82 ± 44.77 ^c^	5256.55 ± 55.59 ^b^	4897.85 ± 75.29 ^a^
SGR/(%/d)	7.25 ± 0.01 ^d^	7.32 ± 0.02 ^e^	7.17 ± 0.01 ^c^	7.11 ± 0.02 ^b^	6.98 ± 0.03 ^a^
FCR	1.15 ± 0.01 ^b^	1.11 ± 0.02 ^a^	1.21 ± 0.01 ^c^	1.26 ± 0.03 ^d^	1.35 ± 0.02 ^e^
SR/%	99.17 ± 1.44 ^a^	100.00 ± 0.00 ^a^	100.00 ± 0.00 ^a^	98.33 ± 1.44 ^a^	98.33 ± 2.89 ^a^

Note: Values in the table are Mean ± SD (*n* = 3). Different superscripts in the same row indicate significant differences (*p* < 0.05). Abbreviations: FBW, final body weight; WGR, weight gain rate; FCR, feed conversion rate; SGR, specific growth rate; SR, survival rate.

**Table 3:** Effects of OLE on the whole-body composition of juvenile *L. vannamei* (%).

Items	Groups				
	OLE0	OLE1	OLE2	OLE4	OLE8
MS	76.56 ± 0.97 ^a^	75.05 ± 1.27 ^a^	76.38 ± 0.17 ^a^	75.41 ± 0.48 ^a^	76.25 ± 1.22 ^a^
CP	69.00 ± 0.05 ^a^	69.92 ± 0.38 ^b^	69.25 ± 0.46 ^ab^	71.42 ± 0.45 ^c^	71.60 ± 0.54 ^c^
CL	12.79 ± 0.14 ^c^	10.53 ± 0.67 ^a^	11.76 ± 0.08 ^b^	12.10 ± 0.09 ^bc^	11.43 ± 0.61 ^b^
CA	12.34 ± 0.07 ^a^	12.50 ± 0.04 ^a^	13.25 ± 0.09 ^a^	12.38 ± 0.32 ^a^	12.78 ± 0.02 ^a^

Note: Values in the table are Mean ± SD (*n* = 3). Different superscripts in the same row indicate significant differences (*p* < 0.05). Abbreviations: MS, moisture; CP, crude protein; CL, crude lipid; CA, crude ash.

**Table 4:** Effects of OLE on serum biochemical indices in juvenile *L. vannamei*.

Items	Groups				
	OLE0	OLE1	OLE2	OLE4	OLE8
TP/(g/L)	53.17 ± 6.51 ^a^	61.79 ± 7.17 ^ab^	64.15 ± 4.09 ^ab^	65.72 ± 5.19 ^b^	70.56 ± 6.30 ^b^
HDL-C/(mmol/L)	1.19 ± 0.02 ^a^	1.46 ± 0.14 ^b^	1.45 ± 0.05 ^b^	1.36 ± 0.12 ^ab^	1.26 ± 0.05 ^a^
LDL-C/(mmol/L)	1.41 ± 0.09 ^c^	1.33 ± 0.05 ^c^	1.27 ± 0.13 ^c^	0.98 ± 0.16 ^b^	0.53 ± 0.06 ^a^
ACP/(U/L)	91.20 ± 2.00 ^a^	118.00 ± 1.39 ^b^	124.00 ± 1.51 ^c^	124.20 ± 1.80 ^c^	131.80 ± 4.81 ^d^
AKP/(U/L)	29.26 ± 1.33 ^a^	42.79 ± 2.09 ^b^	39.50 ± 3.34 ^b^	44.81 ± 3.15 ^b^	44.90 ± 4.29 ^b^
ALT/(U/L)	87.18 ± 2.41 ^a^	84.22 ± 4.47 ^a^	84.48 ± 4.83 ^a^	84.86 ± 8.12 ^a^	87.76 ± 4.79 ^a^
AST/(U/L)	96.55 ± 4.37 ^c^	81.66 ± 3.76 ^b^	78.08 ± 4.54 ^b^	63.28 ± 6.46 ^a^	78.60 ± 1.16 ^b^

Note: Values in the table are Mean ± SD (*n* = 3). Different superscripts in the same row indicate significant differences (*p* < 0.05). Abbreviations: TP, total protein; HDL-C, high-density lipoprotein cholesterol; LDL-C, low-density lipoprotein cholesterol; ACP, acid phosphatase; AKP, alkaline phosphatase; ALT, alanine transaminase; AST, aspartate transaminase.

**Table 5:** Effects of OLE on antioxidant capacity of the hepatopancreas in juvenile *L. vannamei*.

Items	Groups				
	OLE0	OLE1	OLE2	OLE4	OLE8
MDA/(nmol/mL)	12.46 ± 0.10 ^e^	8.77 ± 0.11 ^c^	9.57 ± 0.14 ^d^	5.71 ± 0.12 ^a^	6.44 ± 0.22 ^b^
SOD/(U/mL)	107.90 ± 1.41 ^a^	225.95 ± 3.47 ^e^	179.37 ± 5.46 ^d^	140.09 ± 2.57 ^c^	121.31 ± 2.66 ^b^
GPX/(U/mL)	248.42 ± 14.00 ^a^	495.54 ± 9.51 ^c^	551.92 ± 12.20 ^d^	294.50 ± 4.82 ^b^	246.92 ± 3.46 ^a^
CAT/(U/mL)	68.75 ± 0.96 ^c^	104.08 ± 1.74 ^d^	53.25 ± 1.51 ^b^	54.33 ± 1.83 ^b^	37.73 ± 1.13 ^a^

Note: Values in the table are Mean ± SD (*n* = 3). Different superscripts in the same row indicate significant differences (*p* < 0.05). Abbreviations: MDA, maldodicarboxylic acid; SOD, superoxide dismutase; GPX, glutathione peroxidase; CAT, catalase.

**Table 6:** Effects of OLE on intestinal digestive enzyme activities in juvenile *L. vannamei*.

Items	Groups				
	OLE0	OLE1	OLE2	OLE4	OLE8
LPS/(U/L)	529.89 ± 18.27 ^b^	882.47 ± 11.47 ^d^	730.71 ± 24.02 ^c^	559.37 ± 20.36 ^b^	415.60 ± 5.37 ^a^
AMS/(U/L)	386.84 ± 13.77 ^b^	455.57 ± 9.16 ^d^	439.91 ± 2.29 ^d^	406.09 ± 14.67 ^c^	192.81 ± 5.49 ^a^
TYS/(U/mL)	998.81 ± 60.78 ^a^	2137.21 ± 23.36 ^d^	2165.65 ± 63.70 ^d^	1751.26 ± 36.56 ^b^	1942.86 ± 33.65 ^c^

Note: Values in the table are Mean ± SD (*n* = 3). Different superscripts in the same row indicate significant differences (*p* < 0.05). Abbreviations: LPS, lipase; AMS, amylase; TYS, trypsin.

**Table 7:** Effects of OLE on intestinal species diversity in juvenile *L. vannamei*.

Items	Groups				
	OLE0	OLE1	OLE2	OLE4	OLE8
OTUs	231.33 ± 29.91 ^a^	276.67 ± 26.08 ^a^	259.00 ± 42.44 ^a^	298.33 ± 23.46 ^a^	447.00 ± 103.79 ^b^
Ace	274.47 ± 24.30 ^a^	313.91 ± 22.62 ^a^	312.35 ± 47.56 ^a^	331.63 ± 36.48 ^a^	481.73 ± 103.30 ^b^
Chao1	278.16 ± 9.25 ^a^	306.54 ± 23.02 ^a^	308.12 ± 42.83 ^a^	330.00 ± 40.91 ^a^	471.83 ± 94.81 ^b^
Shannon	2.75 ± 0.69 ^ab^	2.29 ± 0.03 ^a^	2.69 ± 0.24 ^ab^	3.80 ± 0.61 ^c^	3.65 ± 0.73 ^bc^
Simpson	0.70 ± 0.13 ^a^	0.70 ± 0.00 ^a^	0.67 ± 0.01 ^a^	0.78 ± 0.09 ^a^	0.78 ± 0.11 ^a^
Coverage/%	99.95 ± 0.00 ^a^	99.95 ± 0.00 ^a^	99.95 ± 0.00 ^a^	99.97 ± 0.01 ^a^	99.95 ± 0.01 ^a^

Note: Values in the table are Mean ± SD (*n* = 3). Different superscripts in the same row indicate significant differences (*p* < 0.05).

## Data Availability

The data that support the findings of this study are available on request from the corresponding author.

## Supplementary Materials

The following supporting information can be downloaded at https://www.mdpi.com/article/10.3390/ani16152418/s1, Table S1. Nucleotide sequences of the primers used to assay gene expressions by real-time PCR.
