# Supplementary material for: The Effect of Low-Fishmeal Feed Including an Olive Leaf Extract (Olea europaea) Additive on Growth, Intestinal Health and Disease Resistance in Litopenaeus vannamei
Source: Animals (Basel). 2026 Aug 5;16(15):2418. doi: 10.3390/ani16152418 (PMC13464928; doi:10.3390/ani16152418)
Supplement: Supplementary file 1 [file animals-16-02418-s001.zip › Table S1 Nucleotide sequences of the primers used to assay gene expressions by real-time PCR.pdf]

**Table S1 Nucleotide sequences of the primers used to assay gene expressions by real-time PCR**

| Primer | Sequence (5'-3') | Source or GenBank No. |
|---|---|---|
| *β-actin*-F | AATCGTTCGTGACATCAAGGAG | AF300705.2 |
| *β-actin*-R | TTGTAGGTGGTCTCGTGGATG | |
| *toll*-F | CCAGCTTAGAAGACCGGCAA | DQ923424 |
| *toll*-R | GTTGTCCGAGCAGAAGTCCA | |
| *lzm*-F | TGTTCCGATCTGATGTCC | AY170126.2 |
| *lzm*-R | GCTGTTGTAAGCCACCC | |
| *cru a*-F | GGTGTTGGTGGTGGTTTCCC | AY486426.1 |
| *cru a-R* | CAGTCGCTTGTGCCAGTTCC | |
| *imd*-F | CGGCTCTGCGGTTCACAT | FJ592176.1 |
| *imd*-R | CCTCGACCTTGTCTCGTTCCT | |
| *propo*-F | CGGTGACAAAGTTCCTCTTC | Zhao et al [22] |
| *propo*-R | GCAGGTCGCCGTAGTAAG | |

Abbreviations: F, forward; R, reverse; *toll, Toll-like receptors*; *lzm, lysozyme*; *cru a, crustin a*; *imd*, *immune deficiency*; *propo, prophenoloxidase*.
